# A Review of *Salmonella* and Squamates (Lizards, Snakes and Amphisbians): Implications for Public Health

**DOI:** 10.3390/pathogens6030038

**Published:** 2017-08-22

**Authors:** Harriet Whiley, Michael G. Gardner, Kirstin Ross

**Affiliations:** 1 College of Science and Engineering, Flinders University, GPO Box 2100, Adelaide, SA 5001, Australia; Michael.Gardner@flinders.edu.au (M.G.G.); Kirstin.Ross@flinders.edu.au (K.R.); 2 Evolutionary Biology Unit, South Australian Museum, North Terrace, Adelaide, SA 5000, Australia

**Keywords:** public health, zoonosis, lizards, snakes, salmonellosis, *Salmonella*

## Abstract

Globally, there has been an increase in squamates (particularly lizards and snakes) being kept as pets. Additionally, urban spread has resulted in greater human encroachment and interaction with the natural habitat of wild squamates. A potential consequence of increasing human interaction with squamates is the increased potential for disease transfer. This review collates the literature describing clinical salmonellosis cases that were definitively linked to a squamate through testing of the animal and population-based studies which investigate the risk of salmonellosis linked to pet squamates. It was demonstrated that although squamate-acquired salmonellosis accounted for a small percentage of total cases, children under five were at greatest risk, with the clinical manifestations tending to be more severe. In many cases, it was noted that the patient was unaware of the risks associated with keeping squamates and did not practice proper hand hygiene after handling the animals or cleaning cages. This highlights the need for more education focused on informing the general public of ways to reduce the risk of salmonellosis from pet squamates. There is also the need for future research into the role of wild squamates in the spread of human salmonellosis, both directly and indirectly through cross contamination.

## 1. Introduction

*Salmonella* is a significant cause of disease, affecting both humans and animals [1]. It is the causative agent of salmonellosis, a gastrointestinal disease of public health significance [2]. Globally, it is estimated that there are 93.8 million cases of salmonellosis each year [3]. Infrequently, *Salmonella* is also responsible for more invasive diseases, such as bacteraemia with or without metastatic disease, skin and bone infections, urinary tract infections, meningitis and splenic abscess [4,5,6,7,8]. Primarily, *Salmonella* is considered a foodborne pathogen, with contaminated food attributed to 80 million cases of salmonellosis annually [3,9]. However, in the USA it has been estimated the 6% of sporadic salmonellosis cases and 11% of cases in people aged under 21 are caused by reptile and amphibian contact [10]. This estimate was supported by a literature review conducted by Sauteur et al. [11] that examined published studies from 1965 to 2012 describing reptile associated salmonellosis in children aged less than 18 years. A total of 182 cases were identified. The primary reptile associated with gastrointestinal salmonellosis was turtles; however, exposure to iguanas was significantly more prevalent in children with invasive *Salmonella* diseases (septicaemia or meningitis). 

Exotic pets, including lizards and snakes, have become increasingly popular [1,12,13]. *Salmonella* is often detected from captive reptiles and reports of salmonellosis linked to reptile pets are increasing [14]. A study in Malaysia demonstrated that 83.3% of captive lizards (*Iguanidae*, *Agamidae*, *Scincidae*, *Gekkonidae*, *Varanidae*) and 25% of wild lizards (*Agamidae*, *Scincidae*, *Gekkonidae*) were positive for *Salmonella* [15]. The significantly higher (*p* < 0.05) carriage rate in captive lizards compared with wild lizards is supported by a similar study conducted in Germany by Geue and Löschner [16] and could be attributed to horizontal transmission of *Salmonella* from humans and other animals to the captive lizards. In Japan, 66% (47/71) of lizards and 100% (23/23) of snakes from a pet store were positive for *Salmonella* [17]. In Croatia, it was found that 48.4% of captive lizards, and 8.9% of captive snakes belonging to a private owner or the Zagreb Zoo, were positive for *Salmonella* [18]. Thirty nine percent (58/149) of lizards and 29% (31/106) of snakes, housed in zoos and with private keepers in Poland, tested positive for *Salmonella* [19]. Additionally, in Canada, 51% of pet snakes and 48% of pet lizards submitted for autopsy were found to be positive for *Salmonella* and salmonellosis was identified as the cause of death in about one third of the *Salmonella*-positive animals [20]. The incidence of *Salmonella* in the pet trade is likely underreported as few studies have explicitly examined the incidence of this bacterium, despite the increase of snakes and lizards as pets worldwide.

One of the implications of the increasing demand for exotic pets is the potential for international importation of reptiles resulting in disease globalization [21]. In 1996, Sweden no longer required a certificate stating that an animal was *Salmonella*-free prior to importation into the country; consequently, an increase in the incidence of reptile-associated salmonellosis was observed in 1997 [13]. Additionally, a study in the USA found that 80% (88/110) of wild-caught Indonesian Tokay geckos (*Gekko gecko*) imported into the USA were positive for *Salmonella*. This included 14 different serogroups and 17 unique serotypes, several of which demonstrated antibiotic resistance [13].

The presence of *Salmonella* in wild lizards and snakes (squamates) may also be playing a role in human salmonellosis [13]. One consequence of urbanisation resulting in increasing human encroachment into natural ecosystems is a greater potential for interaction between humans and wild animals, leading to greater potential for the transfer of zoonotic pathogens [22]. There have been several studies which have investigated the presence of *Salmonella* in wild lizards and snakes [23,24,25,26]. This includes a study in the Galápagos, Ecuador, that found 62/63 (98%) of land iguanas (*C. subcristatus)* were positive for *Salmonella* [23]. Another study of wild snakes in Poland found that 14/16 (86%) of dead wild European grass snakes (*Natrix natrix*) and smooth snakes (*Coronella austriaca*) were positive for *Salmonella* [25]. In Spain, a study during the spring and summer period, 49% of wild lizards and snakes tested were positive for *Salmonella* [26]. Another study from Australia found that *Salmonella enterica* was present in 83% (50/60) of wild Australian sleepy lizards (*Tiliqua rugosa*). Analysis of the distribution of *Salmonella* genotypes suggested that the bacteria was spread from host to host within the lizards’ social network, rather than exposure to the same environmental source [24]. 

The increase in human interaction with lizards and snakes suggests that they may play an increasingly significant role in the spread of human salmonellosis, particularly with regard to the more invasive infections observed in younger children [10,11,13]. This article examines the current evidence linking human salmonellosis to lizards and snakes. Studies from the last 20 years which confirm a squamate as the source of a human *Salmonella* infection, and population-based studies which examine the likelihood of human *Salmonella* infections as a consequence of exposure to lizards and snakes, are analysed. Trends in *Salmonella* species, squamate species, exposure routes, and patient outcomes are discussed. 

## 2. Results

The population-based studies that examined the likelihood of notified salmonellosis cases to be linked to lizards and snakes are presented in Table 1. The studies are limited to the UK [27], USA [28,29,30,31], and Germany [32]. The most recent study is from the UK, which identifies that over a quarter of salmonellosis cases in children under five years of age are linked to reptile exposure; however, the species of reptiles involved were not examined [27]. The focus on exposure of children is supported by a study from the USA that demonstrated the median age for reptile associated salmonellosis was 11 years old. This study also demonstrated that reptile exposure was attributed to 3.5% of all salmonellosis cases and of these lizards and snakes were attributed to 47% and 20%, respectively [28]. Additionally another study from the USA investigated the source of notified *S.* Marina cases, specifically, and found 81% of patients were infants (less than one year of age) and 88% of these cases reported exposure to iguanas [29]. 

The case reports from the last twenty years which identify and confirm snakes or lizards as a source of human *Salmonella* infection are presented in Table 2. Studies were from Switzerland [33], Germany [34], USA [6,35,36,37,38], Australia [39], UK [7,40,41,42,43], France [44], The Netherlands [8], and Canada [13]. The most commonly-identified lizard or snakes were bearded dragons [34,35,40,45] and iguanas [6,13,36,37,42,43]; although other species included corn snakes [37,44], a water dragon [41], a boa constrictor [38], and a gecko [7]. The cases described in Table 2 demonstrate a range of clinical presentations including gastrointestinal, sinus, blood, brain, bone, urinary tract, and spleen infections and primarily involved young children and immunocompromised patients. In several cases observed in children less than six months of age the *Salmonella* infection was fatal [36,37,41,42]. The most commonly-identified infectious agents were *Salmonella* Marina [6,13,37] and *Salmonella* Poona [13,36,42]. Interestingly, not all patients described direct contact with the lizard or snake, despite an identical isolate from both animal and patient [35,37,40,45], suggesting that indirect contact may play a role in the spread of lizard- and snake-associated salmonellosis. Indirect exposure could also apply to the cases involving young children less than six months of age who are unlikely to have had direct contact with the snake or lizard [7,36,37,41,42,43,45]. This transmission route was speculated by Glick and Sherman [46] who suggested that the mother or another family member acted as the vector transferring the *Salmonella* from the Gecko to the four day old baby. The role of indirect exposure is illustrated by the outbreak involving a potluck dinner which was prepared in the cook’s home and the *Salmonella* serotype responsible for the outbreak was found in her vacuum cleaner and from one of her pet bearded dragons which were kept in an adjacent room [35]. Similarly, in a case from Australia, the patient had no direct contact with the bearded dragon, but the vacuum cleaner contained the same *Salmonella* serotype, suggesting that this could be the route of exposure [45]. A common observation made in the case reports was that patients and the parents of patients were not aware of the risk associated with handling lizards and reported poor hand hygiene practices after handling of snakes and lizards and cleaning of enclosures. It was noted in a case involving a 29 years old male from Switzerland that the patient did no wash or disinfect his hands after handling snakes, feeding them, or cleaning their terrarium [33].

## 3. Discussion

Increasing interaction with snakes and lizards, both as captive pets and through encroachment of their natural ecosystem, may result in an increase in the transmission of salmonellosis [13]. This study demonstrates that snake- and lizard-associated salmonellosis is being reported across the globe. It also highlights the diversity of clinical presentation of *Salmonella* infections associated with snakes and lizards and their potential severity. Children were the primary demographic identified in squamate-associated salmonellosis cases and the clinical manifestations were typically more severe than other cases of salmonellosis. These findings support the US Centres for Disease Control and Prevention (CDC) recommendation that children under the age of five should avoid contact with reptiles and that these animals should not be kept in childcare centres [47]. 

The lack of the general public’s knowledge regarding the dangers associated with lizards and snakes was also highlighted and is supported by the finding of previous studies as one of the main risks for reptile associated salmonellosis [13,37]. In 2003, the US CDC found that only 4/49 US state health departments interviewed required pet store owners to provide information regarding risk of salmonellosis with the purchase of a turtle and no state health department required salmonellosis information to be provided to a person purchasing a lizard of snake [48]. This demonstrates the need for more education aimed at informing snakes and lizards handlers of the associated risk of salmonellosis and the importance of good hand hygiene practices [37]. Good hygiene is particularly important in cases where persons handling snakes and lizards were identified as the potential vector transmitting *Salmonella* to children. Additionally, in cases where there was no direct contact with the lizard or snakes, vacuum cleaners were identified as a potential source. The mechanical disturbance and agitation of settled dust containing microorganisms by a vacuum cleaner can provide a mechanism for the dispersal of bioaerosols [49]. It has been demonstrated vacuum cleaners can disperse bacteria emissions at concentrations as high at 10^5^ bacteria per minute [50]. 

No studies which definitively identified a case of human salmonellosis caused by wild lizards and snakes were identified. However, previous studies have demonstrated the potential for wild lizards and snakes to carry *Salmonella* and, as such, they could potentially be playing a role in human cases [23,24,25,26]. The presence of *Salmonella* in wild lizards and snakes also presents other issues relating to biosecurity and animal health. It has been demonstrated that *Salmonella* is an opportunistic pathogen of lizards and snakes with weakened immune systems [51] and can cause bone infection in snakes [52,53]. There is also the potential for lizards and snakes to transfer *Salmonella* to animal production facilities, such as poultry farms. This could have significant consequences, particularly with the spread of antibiotic-resistant strains between flocks and after disinfection processes [54,55].

## 4. Materials and Methods

**Figure 1:**
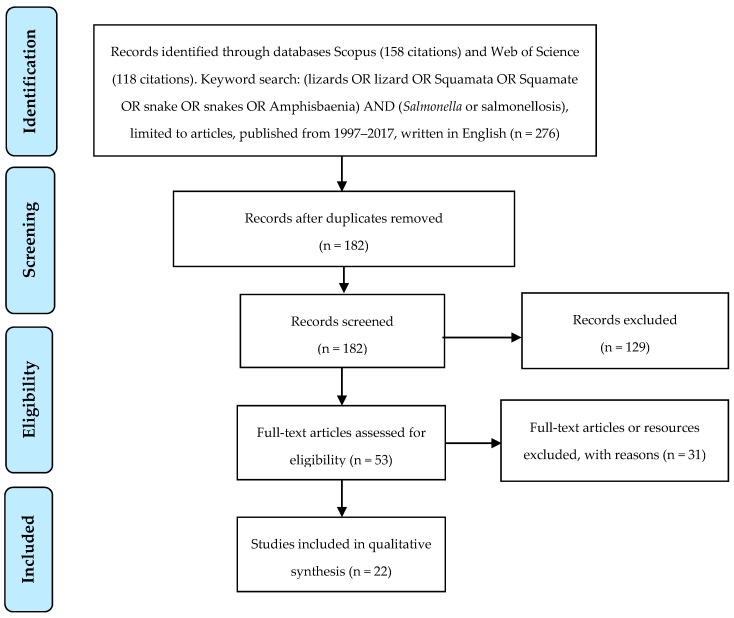
Overview of search methods and articles’ inclusion and exclusion criteria.

The databases Scopus and Web of Science were searched for articles written in English over the last twenty years containing the keywords (*Salmonella* OR salmonellosis) AND (lizards OR lizard OR Squamata OR Squamate OR snake OR snakes OR Amphisbaenia). Figure 1 presents the systematic approach to article inclusion or exclusion. Articles were screened by reading titles and abstracts and initially excluded if they did not refer to human salmonellosis or if they were review articles. Articles were then read in full and excluded if they described a clinical case of *Salmonella* infection linked to a squamate which was not confirmed through testing of the animal. Articles were included if they were population studies investigating squamates as a potential risk for salmonellosis and clinical cases that were definitively linked to a squamate through testing of the animal and comparison of animal and human *Salmonella* isolates. 

## 5. Conclusions

This review demonstrates that salmonellosis associated with lizards and snakes is an emerging global issue of public health concern. Although it only accounts for a small proportion of all salmonellosis cases, the evidence suggests that it predominately affects children under five years of age and the clinical manifestations can be severe. There is a need for greater education aimed at informing people who keep lizards and snakes as pets of the potential risks and the best ways to protect themselves.

## Figures and Tables

**Table 1:** Salmonellosis population-based studies investigating the risks associated with snakes and lizards.

Country	Demographic	Results	Comments	Reference
UK	Children aged under five years with notified salmonellosis in South West of the UK from January 2010 to December 2013.	48 of 175 (27%) *Salmonella* cases had exposure to reptiles.	Reptile associated salmonellosis were 2.5 times more likely to be hospitalized compared to other salmonellosis cases.	[27]
USA	All Minnesotans with notified salmonellosis from 1996 to 2011 were interviewed and those that had been in contact with reptiles were identified.	Of the 8389 sporadic cases of non-typhoidal salmonellosis, 290 (3.5%) reported reptile exposure and 47% of these were identified as lizards, 20% snakes and 14% a combination of reptile types. 60 reptiles tested positive for *Salmonella* and 36 (60%) yielded the same *Salmonella* serotype as the human isolate.	The median age of case patients with reptile exposure was 11 years, 31% were under the age of five years and 67% were under the age of 20 years. The three most common serotypes were Typhimurium (15%), Enteritidis (7%) and subspecies IV serotypes (7%).	[28]
Germany	Notified cases of *Salmonella enterica* serovar Tennessee in children <3 years old from 1 September 2007 to 31 December 2008. For the case control study, a control was a child with notified rotavirus infection in the matching district, frequency matched by age group.	Eighteen cases (16 households) were identified. In eight of 16 case households reptiles were kept. In four of the other households alternative forms of reptile contact were reported. keeping of a reptile and “any reptile contact” were associated with *Salmonella* Tennessee infection (mOR 29.0; 95% CI 3.1±∞ and mOR 119.5; 95% CI 11.7−∞ ).	In two cases the identical strain of *Salmonella* Tennessee was identified from a reptile in the household. Direct contact between child and reptile was denied.	[32]
USA	Cases of *Salmonella enterica* serotype Kingabwa reported to the National *Salmonella* Surveillance System.	Investigation into an outbreak of 6 people in 2005 with the same strain of *S.* Kingabwa found they did not know each other and did not identify any common food or environmental sources. However 4 of the 6 patients had known exposure to lizards (3 water dragons and 1 bearded dragon). The probability of finding this at random is 0.000002.	18 isolates were analyzed using PFGE and 13 were identical. They were isolated from 2001 to 2005 from different states and no common food or environmental source was identified.	[30]
USA	Population-based case-control study was conducted during 2002–2003 in eight sites of the Foodborne Diseases Active Surveillance Network (FoodNet). Included 215 patients with *Salmonella* Newport infection and 1154 healthy community control subjects.	Case patients with pansusceptible (not antibiotic-resistant) infection were also more likely to have a frog or lizard in their household (OR, 2.9 [95% CI, 1.1–7.7]).		[31]
USA	All notified cases of salmonellosis caused by *S.* Marina (32 cases) reported in 1994. Patients were interviewed about demographic information, clinical course, diet, travel history, and contact with reptiles before illness.	Of 28 patients (88%) with reported iguana exposure, only four (14%) touched the reptile, and only 12 respondents (43%) realized that it might have been the source of infection. Seven (32%) of 22 families who owned an iguana at the time of illness continued to own an iguana when contacted a median of 28 weeks later.	Twenty-six (81%) of 32 patients were infants (<1 year of age) and 24 (75%) were male.	[29]

**Table 2:** Studies describing human *Salmonella* infection confirmed to be caused by squamate exposure.

Country	Demographic	Results	*Salmonella* spp.	Squamate	Comments	Reference
Switzerland	A 29 year old man	The same *Salmonella* serotype was isolated from the patient and from the fecal samples collected from 3 of his 5 pet snakes.	*S. enterica* subspecies *diarizonae*, serotype 47: kz35.	Pet snakes (species not reported)	First reported cases of maxillary sinusitis caused by *Salmonella enterica* subspecies *diarizonae* and the third reported cases of *Salmonella* associated sinusitis in the literature.	[33]
Germany	From 2010–2011, 206 households with children <3 years had notified salmonellosis. In 103 households the *Salmonella* was identified as a serovar other than *S.* Typhimurium and *S.* Enteritidis. A total of 79 households were contacted, and almost half (34/79) had at least one reptile in the home. Of the households, 19 were further studied, whereby a total of 36 reptiles were investigated.	In 15 of 19 households, an identical serotype to the human case was confirmed in at least one reptile (including 16 of all 19 bearded dragons examined).	*Salmonella* other than *S.* Typhimurium and *S.* Enteritidis.	Bearded dragon	Altogether, 319 *Salmonella* isolates were investigated and 24 different serovars identified in the reptiles.	[34]
USA	Patients identified through an outbreak investigation by Minnesota department of health after eating the same potluck dinner.	Samples were collected from the home where the dinner had been prepared. The outbreak PFGE subtype of *Salmonella* subspecies IV and *Salmonella enterica* subspecies I, serotype Labadi, were identified from the contents of the vacuum-cleaner bag. *Salmonella* Labadi also was cultured from a cloacal swab of one of the owners pet bearded dragons.	*Salmonella* IV 6,7:z4,z24.	Bearded dragon	Sixty-six of 73 persons who had consumed a potluck dinner were interviewed, of these 19 cases were identified.	[35]
Australia	A four month old girl	Same serotype of *Salmonella* was isolated from the child and environmental samples collected from the terrarium (lizard faeces, drinking water, bark, swabs of the terrarium environment) of the family‘s four-year-old pet eastern bearded dragon.	*S. enterica* serotype Rubislaw.	Bearded dragon	The girl was admitted to the emergency department with salmonellosis.	[45]
UK	A 67 year old female	*Salmonella* isolated concurrently from a hospital inpatient and a pet bearded dragon lizard. Isolates were identical by biochemical profiling and pulsed-field gel electrophoresis.	*S. enterica* serotype Apapa.	Bearded dragon	Patient reported no direct contact with the lizards and her son cleaned the tank	[40]
France	A 10 month old boy	Same subspecies of *Salmonella* was isolated from the patient and from the stool of the corn snake.	*S. enterica* subspecies *arizonae.*	Corn snake	*Salmonella* osteoarticular infection (septic arthritis of the hip).	[44]
The Netherlands	A 17 year old girl	Same serotype *Salmonella* was isolated from patient clinical samples and reptile fecal samples.	*S. enterica* serotype Telelkebi.	Salamander and bearded dragon	Splenic abscess cause by *Salmonella.*	[8]
USA	Two patients who received platelet donations from a donor with a pet boa constrictor.	A stool sample from the boa grew same *Salmonella* serotype isolated from the platelets.	*S. enterica* serotype Enteritidis.	Boa constrictor	*Salmonella* sepsis caused by a platelet transfusion from a donor with a pet snake.	[38]
UK	A two-months old	S. chameleon was identified from the patient cerebrospinal fluid. *S.* Chameleon is of subspecies IV. *Salmonella* Marina, another member of subspecies IV was detected from the gecko tank.	*S.* Chameleon.	Gecko	*Salmonella* meningitis.	[7]
UK	A three week old baby and the baby‘s mother	Clinical isolates and isolates taken from the water dragons drinking water and a piece of wood in the cage were positive for *S.* Rubislaw.	*S. enterica* serotype Rubislaw.	Water dragon	*Salmonella* meningitis in the neonate was fatal.	[41]
UK	A four month old baby	Same serotype *Salmonella* was identified from the brain tissue during autopsy and from the iguana.	*S. enterica* serovar Poona.	Iguana	*Salmonella* meningitis was fatal.	[42]
USA	A three week old boy	Stool samples from both the patient and the family pet iguana were positive for same *Salmonella* serotype.	*S. enterica* serotype IV 44.	Iguana	The iguana was moved to a relative‘s house. One month later the infant spent two days at the relatives house and once again developed salmonellosis and the stool sample again tested positive for *Salmonella* IV 44: _z4,z23-_.	[37]
	A six year old boy	Stool cultures from both the child and the pet corn snakes yielded the same serotype of *Salmonella*.	*S. enterica* serotype Typhimurium.	Corn snake		
	A five months old boy	Culture of a heart blood sample from the patient and stool samples from the iguana were positive for the same serotype of *Salmonella*.	*S. enterica* serotype Marina.	Iguana	This case was fatal. The iguana had no direct contact with the infant.	
USA	A three week old boy	It was demonstrated using restriction fragment length polymorphism (RFLP) analysis and ribotyping that the *Salmonella* isolates collected from patient and the iguana were identical.	*S. enterica* serotype Poona.	Iguana	This case was fatal.	[36]
Canada	Notified cases of salmonellosis linked to lizards from 1994 to 1996	Cases of human salmonellosis with a firmly established epidemiological link to a pet lizard. In epidemiological studies related to human infection, the same *Salmonella* serotypes observed in humans were also identified in exotic pets.	*S. enterica* serotype Poona.	Iguana	One case.	[13]
			*S. wassenaar* subsp. IV.	Iguana	One case and one outbreak (a family involving five cases.	
			*S. enterica* serotype Montevideo.	Iguana	One case.	
			*S. enterica* serotype Marina.	Iguana	Four cases.	
UK	An 11 day old boy	*Salmonella* was isolated from the stool of one child and also from an iguana kept in the home as a pet.	*S.* Chameleon.	Iguana		[43]
USA	A 40 year old woman	Cultures of urine and blood from patient and the stool of the pet iguana were positive for *Salmonella.*	*S. enterica* serotype Marina.	Iguana	Patient was suffering *Salmonella* urinary tract infection.	[6]
